# Supplementary material for: Cohort Profile Update: The HUNT Study, Norway
Source: Int J Epidemiol. 2022 May 17;52(1):e80–91. doi: 10.1093/ije/dyac095 (PMC9908054; doi:10.1093/ije/dyac095)
Supplement: dyac095_Supplementary_Data [file dyac095_supplementary_data.zip › dyac095_Supplementary_Data/ije-2021-10-1496-File007.pptx]

## Slide 1
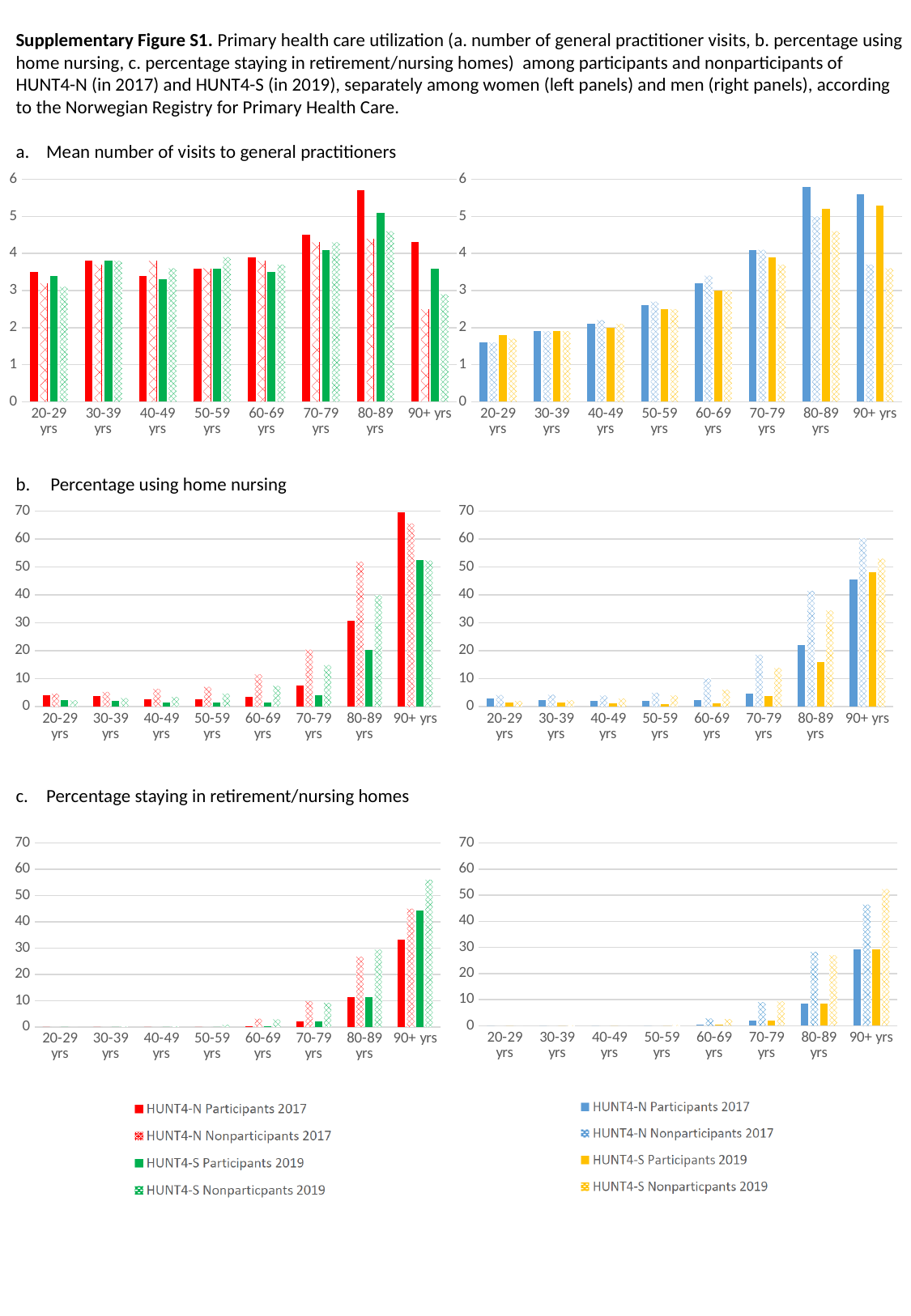

Supplementary Figure S1. Primary health care utilization (a. number of general practitioner visits, b. percentage using
home nursing, c. percentage staying in retirement/nursing homes) among participants and nonparticipants of
HUNT4-N (in 2017) and HUNT4-S (in 2019), separately among women (left panels) and men (right panels), according
to the Norwegian Registry for Primary Health Care.
Mean number of visits to general practitioners
 Percentage using home nursing
Percentage staying in retirement/nursing homes
### Chart
| Category | HUNT4-N Participants | HUNT4-N Nonparticipants | HUNT4-S Participants | HUNT4-S Nonparticpants |
|---|---|---|---|---|
| 20-29 yrs | 3.5 | 3.2 | 3.4 | 3.1 |
| 30-39 yrs | 3.8 | 3.7 | 3.8 | 3.8 |
| 40-49 yrs | 3.4 | 3.8 | 3.3 | 3.6 |
| 50-59 yrs | 3.6 | 3.6 | 3.6 | 3.9 |
| 60-69 yrs | 3.9 | 3.8 | 3.5 | 3.7 |
| 70-79 yrs | 4.5 | 4.3 | 4.1 | 4.3 |
| 80-89 yrs | 5.7 | 4.4 | 5.1 | 4.6 |
| 90+ yrs | 4.3 | 2.5 | 3.6 | 2.9 |
### Chart
| Category | HUNT4-N Participants | HUNT4-N Nonparticipants | HUNT4-S Participants | HUNT4-S Nonparticpants |
|---|---|---|---|---|
| 20-29 yrs | 1.6 | 1.6 | 1.8 | 1.7 |
| 30-39 yrs | 1.9 | 1.9 | 1.9 | 1.9 |
| 40-49 yrs | 2.1 | 2.2 | 2.0 | 2.1 |
| 50-59 yrs | 2.6 | 2.7 | 2.5 | 2.5 |
| 60-69 yrs | 3.2 | 3.4 | 3.0 | 3.0 |
| 70-79 yrs | 4.1 | 4.1 | 3.9 | 3.7 |
| 80-89 yrs | 5.8 | 5.0 | 5.2 | 4.6 |
| 90+ yrs | 5.6 | 3.7 | 5.3 | 3.6 |
### Chart
| Category | HUNT4-N Participants | HUNT4-N Nonparticipants | HUNT4-S Participants | HUNT4-S Nonparticpants |
|---|---|---|---|---|
| 20-29 yrs | 4.1 | 4.6 | 2.4 | 2.3 |
| 30-39 yrs | 3.7 | 5.3 | 2.0 | 3.1 |
| 40-49 yrs | 2.8 | 6.4 | 1.4 | 3.5 |
| 50-59 yrs | 2.7 | 7.1 | 1.6 | 4.7 |
| 60-69 yrs | 3.4 | 11.6 | 1.4 | 7.5 |
| 70-79 yrs | 7.5 | 20.4 | 4.2 | 14.9 |
| 80-89 yrs | 30.7 | 51.9 | 20.4 | 39.9 |
| 90+ yrs | 69.5 | 65.6 | 52.4 | 52.3 |
### Chart
| Category | HUNT4-N Participants | HUNT4-N Nonparticipants | HUNT4-S Participants | HUNT4-S Nonparticpants |
|---|---|---|---|---|
| 20-29 yrs | 2.9 | 4.2 | 1.4 | 2.1 |
| 30-39 yrs | 2.4 | 4.3 | 1.4 | 2.3 |
| 40-49 yrs | 2.2 | 4.0 | 1.1 | 2.9 |
| 50-59 yrs | 2.0 | 5.0 | 0.9 | 4.0 |
| 60-69 yrs | 2.4 | 10.1 | 1.3 | 6.0 |
| 70-79 yrs | 4.6 | 18.6 | 3.9 | 13.8 |
| 80-89 yrs | 22.1 | 41.4 | 16.0 | 34.4 |
| 90+ yrs | 45.6 | 60.3 | 48.2 | 52.9 |
### Chart
| Category | HUNT4-N Participants | HUNT4-N Nonparticipants | HUNT4-S Participants | HUNT4-S Nonparticpants |
|---|---|---|---|---|
| 20-29 yrs | 0.0 | 0.0 | 0.0 | 0.0 |
| 30-39 yrs | 0.0 | 0.0 | 0.0 | 0.1 |
| 40-49 yrs | 0.0 | 0.0 | 0.0 | 0.1 |
| 50-59 yrs | 0.0 | 0.0 | 0.0 | 0.8 |
| 60-69 yrs | 0.4 | 3.1 | 0.2 | 2.9 |
| 70-79 yrs | 2.2 | 9.8 | 2.0 | 9.1 |
| 80-89 yrs | 11.4 | 26.7 | 11.3 | 29.4 |
| 90+ yrs | 33.3 | 45.0 | 44.3 | 56.0 |
### Chart
| Category | HUNT4-N Participants | HUNT4-N Nonparticipants | HUNT4-S Participants | HUNT4-S Nonparticpants |
|---|---|---|---|---|
| 20-29 yrs | 0.0 | 0.0 | 0.0 | 0.0 |
| 30-39 yrs | 0.0 | 0.0 | 0.0 | 0.1 |
| 40-49 yrs | 0.0 | 0.0 | 0.0 | 0.0 |
| 50-59 yrs | 0.0 | 0.0 | 0.0 | 0.2 |
| 60-69 yrs | 0.4 | 2.8 | 0.3 | 2.5 |
| 70-79 yrs | 1.9 | 9.0 | 2.0 | 9.3 |
| 80-89 yrs | 8.6 | 28.2 | 8.6 | 27.0 |
| 90+ yrs | 29.1 | 46.4 | 29.2 | 52.3 |
