# Supplementary material for: Cohort Profile Update: The HUNT Study, Norway
Source: Int J Epidemiol. 2022 May 17;52(1):e80–91. doi: 10.1093/ije/dyac095 (PMC9908054; doi:10.1093/ije/dyac095)
Supplement: dyac095_Supplementary_Data [file dyac095_supplementary_data.zip › dyac095_Supplementary_Data/ije-2021-10-1496-File009.docx]

**Supplementary Table S2.** Relative probability of participation in HUNT4-N according to characteristics at participation in HUNT3.^a^

|  |  |  | Women | |  | Men | |
| --- | --- | --- | --- | --- | --- | --- | --- |
| Characteristics at participation in HUNT3  (2006-08) |  |  | HUNT4-N participants, *n* (%)^b^ | Relative probability^c^ (95% CI) |  | HUNT4-N participants, *n* (%)^b^ | Relative probability^c^ (95% CI) |
| Age (years) |  |  |  |  |  |  |  |
| 19-29 |  |  | 1442 (54.5) | 1.00 (reference) |  | 920 (49.3) | 1.00 (reference) |
| 30-39 |  |  | 3029 (75.6) | 1.39 (1.33-1.44) |  | 1923 (67.4) | 1.37 (1.30-1.44) |
| 40-49 |  |  | 4337 (79.6) | 1.46 (1.41-1.52) |  | 3390 (74.3) | 1.51 (1.43-1.58) |
| 50-59 |  |  | 4802 (80.0) | 1.47 (1.41-1.52) |  | 4211 (77.7) | 1.58 (1.50-1.65) |
| 60-69 |  |  | 3670 (71.6) | 1.31 (1.26-1.37) |  | 3193 (68.3) | 1.39 (1.32-1.46) |
| 70-79 |  |  | 1459 (47.3) | 0.87 (0.82-0.91) |  | 1106 (41.5) | 0.84 (0.79-0.90) |
| ≥80 |  |  | 217 (15.1) | 0.28 (0.24-0.31) |  | 120 (12.0) | 0.24 (0.20-0.29) |
| Marital status |  |  |  |  |  |  |  |
| Married or registered partner |  |  | 11 722 (75.6) | 1.00 (reference) |  | 9778 (68.7) | 1.00 (reference) |
| Not married or registered partner |  |  | 3801 (64.4) | 0.90 (0.88-0.92) |  | 3489 (59.7) | 0.86 (0.84-0.89) |
| Divorced, separated or widow(er) |  |  | 3422 (54.2) | 0.87 (0.85-0.89) |  | 1583 (54.0) | 0.82 (0.80-0.85) |
| Constituency type |  |  |  |  |  |  |  |
| Urban |  |  | 6758 (67.6) | 1.00 (reference) |  | 5043 (64.5) | 1.00 (reference) |
| Rural |  |  | 11 599 (70.1) | 1.03 (1.01-1.04) |  | 9438 (65.9) | 1.02 (1.00-1.04) |
| Body mass index (kg/m^2^) |  |  |  |  |  |  |  |
| <25.0 |  |  | 7551 (69.8) | 1.00 (reference) |  | 3553 (60.5) | 1.00 (reference) |
| 25.0-29.9 |  |  | 7267 (70.1) | 1.00 (0.99-1.02) |  | 8127 (68.0) | 1.05 (1.03-1.07) |
| ≥30.0 |  |  | 4072 (64.0) | 0.92 (0.90-0.94) |  | 3127 (62.1) | 0.95 (0.92-0.97) |
| Good or very good self-rated health |  |  |  |  |  |  |  |
| Yes |  |  | 13 943 (72.8) | 1.00 (reference) |  | 11 726 (68.1) | 1.00 (reference) |
| No |  |  | 4421 (58.1) | 0.86 (0.84-0.88) |  | 2807 (53.4) | 0.85 (0.83-0.88) |
| Daily smoking |  |  |  |  |  |  |  |
| Never |  |  | 9551 (69.3) | 1.00 (reference) |  | 7627 (69.8) | 1.00 (reference) |
| Former |  |  | 5763 (72.4) | 0.99 (0.97-1.00) |  | 5071 (62.2) | 0.94 (0.92-0.96) |
| Current |  |  | 3185 (61.5) | 0.81 (0.79-0.83) |  | 1829 (54.1) | 0.77 (0.74-0.79) |
| History of current or previous disease (self-reported) |  |  |  |  |  |  |  |
| Coronary heart disease (myocardial infarction or angina pectoris) |  |  | 18 544 (69.4) |  |  |  |  |
| No |  |  | 18 544 (69.4) | 1.00 (reference) |  | 13 965 (66.4) | 1.00 (reference) |
| Yes |  |  | 410 (39.3) | 0.83 (0.78-0.89) |  | 896 (44.9) | 0.85 (0.81-0.89) |
| Stroke |  |  |  |  |  |  |  |
| No |  |  | 18 657 (68.9) | 1.00 (reference) |  | 14 564 (65.3) | 1.00 (reference) |
| Yes |  |  | 297 (44.9) | 0.83 (0.77-0.90) |  | 297 (40.7) | 0.78 (0.72-0.84) |
| Asthma |  |  |  |  |  |  |  |
| No |  |  | 16 762 (68.8) | 1.00 (reference) |  | 13 287 (64.9) | 1.00 (reference) |
| Yes |  |  | 2192 (64.9) | 0.95 (0.93-0.97) |  | 1574 (61.2) | 0.96 (0.93-0.99) |
| COPD or emphysema |  |  |  |  |  |  |  |
| No |  |  | 18 478 (68.9) | 1.00 (reference) |  | 14 508 (65.3) | 1.00 (reference) |
| Yes |  |  | 473 (50.8) | 0.78 (0.74-0.83) |  | 352 (43.3) | 0.76 (0.70-0.82) |
| Diabetes |  |  |  |  |  |  |  |
| No |  |  | 18 387 (69.0) | 1.00 (reference) |  | 14 273 (65.3) | 1.00 (reference) |
| Yes |  |  | 566 (51.6) | 0.87 (0.83-0.92) |  | 586 (50.2) | 0.83 (0.79-0.88) |
| Cancer |  |  |  |  |  |  |  |
| No |  |  | 18 046 (69.1) | 1.00 (reference) |  | 14 343 (65.6) | 1.00 (reference) |
| Yes |  |  | 907 (55.1) | 0.90 (0.86-0.93) |  | 518 (44.8) | 0.83 (0.78-0.88) |
| Sought health care for mental health problems |  |  |  |  |  |  |  |
| No |  |  | 14 906 (69.6) | 1.00 (reference) |  | 12 850 (65.4) | 1.00 (reference) |
| Yes |  |  | 3446 (65.3) | 0.92 (0.90-0.93) |  | 1617 (61.5) | 0.91 (0.89-0.94) |
| Visit to a general practitioner during the last 12 months |  |  |  |  |  |  |  |
| No |  |  | 3163 (70.6) | 1.00 (reference) |  | 4031 (68.5) | 1.00 (reference) |
| Yes |  |  | 15 792 (67.9) | 1.00 (0.98-1.02) |  | 10 830 (63.1) | 0.99 (0.97-1.01) |
| Admitted to hospital during the last 12 months |  |  |  |  |  |  |  |
| No |  |  | 16 828 (69.2) | 1.00 (reference) |  | 13 500 (65.8) | 1.00 (reference) |
| Yes |  |  | 2127 (61.8) | 0.94 (0.92-0.96) |  | 1362 (54.2) | 0.89 (0.86-0.92) |
|  |  |  |  |  |  |  |  |

^a^ In total, 18 956 (68.3%) of 27 754 female HUNT3 participants and 14 863 (64.5%) of 23 046 male HUNT3 participants participated in HUNT4; the remaining HUNT3 participants either died or moved out of Nord-Trøndelag between the HUNT3 and HUNT4 surveys or were invited but did not participate in HUNT4.

^b^ Percentage among the total number of HUNT3 participants in the category

^c^ Relative probabilities of participation, adjusted for age (in 10-year categories) at participation in HUNT3
