# Supplementary material for: Cohort Profile Update: The HUNT Study, Norway
Source: Int J Epidemiol. 2022 May 17;52(1):e80–91. doi: 10.1093/ije/dyac095 (PMC9908054; doi:10.1093/ije/dyac095)
Supplement: dyac095_Supplementary_Data [file dyac095_supplementary_data.zip › dyac095_Supplementary_Data/ije-2021-10-1496-File014.docx]

**Supplementary Table S7.** Examples on how HUNT data have been used across a range of health topics since the original cohort profile was published in 2013.

| Topic | Finding/contribution | PubMed IDentifier (PMID) |
| --- | --- | --- |
| Airways | Influenced change in the international COPD classification (ABCD).  Validated Global Lung Initiative reference values, now included in Norway. Diagnostic criteria for COPD changed accordingly.  Updated Norwegian prevalence data and trends of COPD  Certain comorbidity clusters, as identified using machine learning software, are associated with increased mortality and risk of severe exacerbations in COPD (under review).  A century of increasing lung function and its implications for the diagnosis of lung disease: Results from 243,465 European adults across ten population-based studies  Contribution to international consortia on lung function reference values and lung cancer risk factors | **23611880**  27824594 30180485  29724393  22743675  29617726 |
| Biometrics | A century of trends in adult human height | 27458798 |
| Bone health | Osteoporosis is associated with increased mortality in individuals with COPD.  Underuse of anti-osteoporotic treatment in persons with high fracture risk  The FRAX risk score without bone mineral density predicts hip fractures reasonably well.  HUNT- data included in ongoing update of FRAX (FRAX2). | 31709837  29774403  28668994 |
| Cardiovascular system (CVD):  Blood pressure | Worldwide trends in blood pressure from 1975 to 2015  Contributions of mean and shape of blood pressure distribution to worldwide trends and variations in raised blood pressure | 27863813  29579276 |
| CVD:  Atrial Fibrillation | Estimated cardiorespiratory fitness inversely associated with AF  Higher physical activity levels and fitness associated with lower risk of CVD and all-cause mortality  Physical activity modifies AF risk in obese | 31246716    32047884    29939081 |
| CVD: Stroke | Albuminuria is a risk factor for ischemic stroke.  The risk of stroke depends on the metabolic consequences of obesity. | 32359353  34281375 |
| Cardiorespiratory fitness | Validated nonexercise model of fitness for prediction of mortality and morbidity.  Provided data on the link between fitness and coronary disease.  New data linking fitness to cardiac structure and function.  Creation and validation of Physical Activity Intelligence index (PAI) to motivate people to become and mainstay physical healthy.  Physical activity has protective effect on depression after myocardial infarction. | 24576863    30496487  31986991  32971113  27866655  27984009    26302141 |
| Cardiovascular:  Echocardiography | Largest normal reference ranges for advanced echocardiography.    Cardiac function of healthy individuals is impaired by risk factors for cardiac disease.  Established how cardiac geometry influence measures of cardiac function.  Evaluated important factors for test-retest variation of echocardiographic measurements  Creation of optimally matched control groups for evaluation of cardiotoxicity in cancer patients.    Creation of optimally matched control groups for evaluation of cardiac dysfunction diabetic patients.  Depression symptoms influence cardiac function. | 19946115  20581050  31544286  21247733    32154940  32978265  33960012  19959533  27038515  26897666  26169610  26948543  25350248  26925243 |
| Cardiovascular:  General | Cardiovascular outcome is impaired by symptoms of depression and anxiety.  Diabetes influence mortality different in men and women.  Reduced cardiovascular mortality in diabetic patients between HUNT1 and HUNT3.  Established novel genes important for cardiac diseases.  Pregnancy complications predicts cardiovascular diseases.  Cardiac biomarkers predict cardiovascular prognosis. | 24057077  25044493  17947212  18595902  24633158  29290336  30596987  31188397  27815376  26294790  30996050 |
| Chronic pain and sleep | Insomnia symptoms are associated with increased risk of chronic pain and pain-related disability. A physically active lifestyle may to some extent compensate the adverse effect of insomnia symptoms on risk of chronic pain. | 24293504  28744933  29699540  31801790 |
| Dementia | Current and future prevalence estimates of mild cognitive impairment, dementia, and its subtypes  Physical performance across the cognitive spectrum and between dementia subtypes  Temporal changes in cardiorespiratory fitness and risk of dementia incidence and mortality  C-reactive protein, blood pressure and chronic kidney disease as risk factors for incident dementia | 33427745  33798998  31677775  29387136  28569205  31299931 |
| Diabetes | Worldwide trends in diabetes since 1980  Low C-peptide and high glutamic acid decarboxylase autoantibody levels predict progression to insulin dependence in LADA  High physical activity level is associated with reduced risk of LADA in individuals without high-risk HLA genetic susceptibility  Overweight interacts with HLA high-risk genotypes but also with genes associated with type 2 diabetes in the promotion of LADA  The validity of FINDRISC and the risk of diabetes among people with FINDRISC ≥15 is lower than assumed in national guidelines  A subset of individuals fulfilling diagnostic criteria for type 2 diabetes display transient signs of autoimmunity preceding diagnosis | 27061677  34318969  32835373  31125083  31803483  30327361 |
| Eating disorders | Maternal eating disorders are associated with adverse obstetric outcomes. | 30189108 |
| Family studies | Parental chronic musculoskeletal pain is associated with increased risk of chronic musculoskeletal pain in the adult offspring. This association is to some extent modified by offspring lifestyle factors, such as physical activity and obesity.  Intergenerational transmission of overweight and obesity in HUNT families | 31904500  29704885  27082110  25096408  30341129  27851798 |
| Gastroenterology | Greatly increased prevalence of gastro-esophageal reflux disease (GORD).  Improvement of GORD with weight loss and tobacco smoking cessation.  No increased mortality with GORD. | 22190483    23358462  24322837  27789657 |
| Headache | Intracranial abnormalities more common in headache sufferers  Migraine is not a a predictor of increased mortality  Previous mild head injuries: Headache more common  Caesarean section and the association with migraine  Time trends: Decreasing prevalence of migraine and MOH  The HUNT4 questionnaire is a valid tool to identify persons with migraine  Elevated CRP increased the risk of chronic migraine | 25896482  26115666 26634833 33208331 32160857  31195960  32503410 |
| Health behavior | Multiple lifestyle behaviors and mortality | 28068991 |
| Hearing | Several cardiovascular risk factors are weakly associated with hearing loss  Family status affects hearing loss mortality  Otitis media in childhood is associated with dizziness and reduced hearing in adulthood  Hearing loss in childhood is related to educational attainment and mental health in adulthood  Sociodemographic factors affect the use of hearing aids  The prevalence of hearing impairment has decreased in Norway the last two decades partly due to increased education, less occupational noise exposure, ear infections and smoking.  The use of personal music players has increased but normal use is not associated with 20-year progression in hearing. | 26642893    30463047  30998545  26335289  30736854  30946138  27429594  33509127 32541261  34181492 |
| MRI/neuroimaging | Incidental intracranial findings and their clinical impact  Marked effects of intracranial volume correction methods on sex differences in neuroanatomical structures  Perimenopausal hormone therapy is associated with regional sparing of the CA1 subfield  How does the accuracy of intracranial volume measurements affect normalized brain volumes? | 26950220  26217172  26130062  25857759 |
| Obesity | Trends in adult body-mass index in 200 countries from 1975 to 2014  Worldwide trends in body-mass index, underweight, overweight, and obesity from 1975 to 2016  Rising rural body-mass index is the main driver of the global obesity epidemic in adults  Social and spatial patterns of obesity diffusion over three decades  Central obesity is associated with lower intake of whole-grain bread, less frequent breakfast and lunch and more frequent nightly meals, when adjusted for age, sex and multiple testing. | 27115820  29029897  31068725  24138786  24833275 |
| Occupational health | Farmers' mental health: A longitudinal sibling comparison  Disability pension and symptoms of anxiety and depression: a prospective comparison of farmers and other occupational groups.  Health and unemployment: 14 years of follow-up on job loss | 27636024  26525724  26715474 |
| Oral health | Prevalence data and trends of dental caries in the Norwegian population.  Inequalities in dental service utilization.  Oral health-related quality of life in older adults.  Classification and current prevalence of periodontal disease among Norwegian adults. | 22779374    23012325  21226856  34101228 |
| Pregnancy and maternal cardiovascular health | Conventional cardiovascular risk factors are important targets for cardiovascular prevention in women with hypertensive disorders of pregnancy.  Adding pregnancy complications to an established CVD risk prediction model made no major improvements to CVD prediction.  Parity is associated with lasting reduction in blood pressure.  The association between preterm delivery and CVD is not explained by commonly measured cardiovascular risk factors. | 31188397      30596987      29980890    33022746 |
| Public health | Trends in disability-free life expectancy (DFLE) from 1995 to 2017 in the older Norwegian population  Employment, behavioral and psychosocial factors associated with geographical inequalities | 33908292  32861971 |
| Social epidemiology | Global effects of income and income inequality on adult height  Socioeconomic position, multimorbidity and mortality  Socioeconomic inequalities in the prevalence of complex multimorbidity | 28199042  32858852  32546494 |
| Thyroid function | Changes in the prevalence of hypothyroidism  Autoimmune diabetes, but not type 2 diabetes, is associated with increased prevalence of hypo- and hyperthyroidism and TPO antibodies  Associations of thyroid function with coronary heart disease, fractures, depressive symptoms and dementia: contributions to the Thyroid Studies Collaboration consortium | 23975540  26583583  25893284, 26010634, 28482002, 33154486, 34491268 |
